# Circumpolar health – what is next?

**DOI:** 10.3402/ijch.v72i0.20713

**Published:** 2013-08-05

**Authors:** Kue Young

**Affiliations:** Dalla Lana School of Public Health, University of Toronto, Toronto, Canada

It is a tremendous honour for me to be speaking at a plenary session on the first day of this 15th International Congress of Circumpolar Health. Unlike the previous speaker, the youthful Dr. Neil Murphy, who gets to speak about the past, this old-timer has been tasked to talk about the future.

First, a couple of personal notes: I first attended ICCH-6, held in Anchorage, Alaska, in 1984. I can proudly claim that I have not missed a single congress since. I therefore feel that it is my duty to caution members of the audience who are here for the first time. The ICCH is habit forming, and it can actually alter lives!

I will, of course, not talk totally about the future, since my guess is as good as yours, and who will hold me responsible years from now when my prediction turns out not to be true? What I will talk about are:

- Who are circumpolar peoples?
- How has our population changed?
- Do we enjoy the same health?
- Why are we unequal?
- What are our health care challenges? and
- What can the circumpolar health “community” do?

## The circumpolar world

**Fig. 1:**
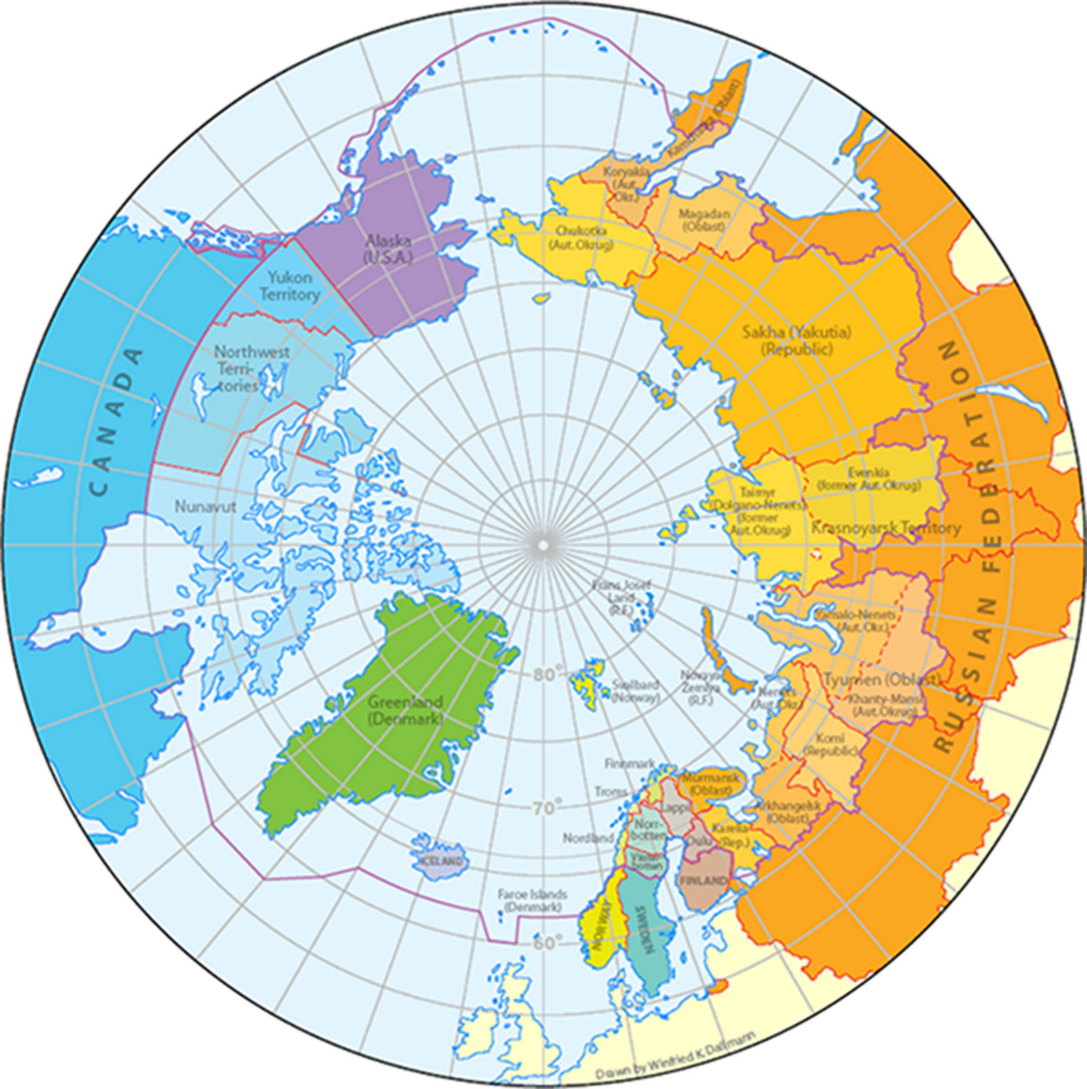
Circumpolar countries and regions.

We have all seen a map of the world as seen from somewhere atop the North Pole (Fig. 1). You will be surprised that many people, including those who are knowledgeable in global health affairs, have never seen the world presented like that. Perhaps the Arctic Ocean in the 21st century is like the Mediterranean in the 1st century. For the sake of argument, let us just say there are 8 Member States of the Arctic Council, and within these, there are some 27 political-administrative regions, the boundaries of which are often in flux, and some may disappear altogether from time to time. Within these 27 regions, there are some 10 million people, belonging to diverse cultures and speaking many tongues. There are indigenous people, and there are migrants both old and new. The North today is very much part of the global economy. It may be remote, but it is no longer isolated.

We tend to know the North in our own backyard best, and often forget that there are other “Norths.” That is why we have “circumpolar” this and “circumpolar” that. If we do not get anything out of these triennial jamborees, we should at the very least recognise the tremendous diversity in many ways, not just across circumpolar regions but also within regions.

Here are some generalisations that you may hear from people speaking about “their North”:

- “The North is numerically small relative to the country as a whole” – well, yes and no. Regions such as Alaska and the northern territories of Canada account for less than 0.5% of the population of the United States and of Canada, respectively, while Greenland and Faroe Island's population is each only about 1% that of Denmark. In contrast, across Eurasia, the population of the northern regions of Norway, Sweden, Finland and Russia range from 5 to 12% of their respective total national population.
- “The North is nothing but a vast expanse of nothing” – it is true that there are very few people occupying a gigantic landmass. For example, Nunavut has only 0.02 persons per km^2^. However, Faroe Islands’ population density is 35 persons per km^2^. You will be surprised to know that most northerners actually live in cities. Within regions, the proportion of the population that live in cities may account from 20 to 70% of the total regional population. While there are no cities having more than 25,000 inhabitants in northern Canada or Greenland, there are large ones in the 50,000 to 150,000 range in northern Scandinavia, and even larger cities can be found in the Russian North, for example, Arkhangelsk (>350,000). Alaska's Anchorage (280,000) is of course a northern metropolis.
- “The North is all about indigenous people” – this is largely true for Nunavut and Greenland, where over 85% of the population are Inuit. In the Northwest Territories, indigenous people account for about 50% of the population; in the Yukon it is 25%, and in Alaska, 20%. In Russia, indigenous people are a sizable minority in several of the autonomous regions: Chukotka (30%), Koryakia (40%), Taymyr (25%) and Nenets (20%). Accurate figures are not available in Scandinavia, but the Sami are likely less than 10% of the population of the northernmost counties, with the possible exception of Finnmark in Norway, which may be higher. Regardless of their numbers or share of the total regional population, it is true that indigenous people experience substantial health disparities relative to other populations in most regions.
- “The North is poor” – if we use the per capita Gross Domestic Product as the yardstick, then most northern regions are not much worse off than their countries as a whole, and the per capita GDP of some regions are actually several times higher, especially those regions where there are large-scale oil and gas developments, for example, the Khanty-Mansi, Yamalo-Nenets and Nenets autonomous regions in Russia. However, how the rich proceeds from natural resources development are distributed and how they actually benefit Northerners is another matter. Within the North, where individual-level socioeconomic indicators are available, indigenous people tend to fare worse than non-indigenous people in most regions.
- “The North is stagnating” – when referring to the rise and fall of population, some regions are experiencing significant population growth, especially Nunavut, Alaska, Iceland, Khanty-Mansi, Northwest Territories and Yamalo-Nenets, due to a variety of reasons, including high fertility and/or high in-migration spurred on by economic development. At the other end are most of the northern regions of Russia (except the 2 mentioned above), which have suffered substantial de-population, with some regions such as Magadan having lost as much as half of their population over a 20-year period.

## Health disparities

The people in the circumpolar North do not all enjoy the same health. There are substantial disparities among countries and regions, and within regions. If we compare circumpolar countries, and using something such as the United Nations Human Development Index, then it is clear that Russia lags far behind the others, ranking 65th in the world in 2010, whereas the other Arctic States are all within the top 20.

Circumpolar regions basically fall into 4 groups:

- The Nordic countries – these do best in every health indicator, and there is generally little difference between north and south, or between indigenous and non-indigenous people;
- Alaska, Yukon and the Northwest Territories – their health status is comparable to, or even better than, the national average of the United States and Canada; however, within these regions, there are substantial disparities between indigenous and non-indigenous people;
- Greenland and Nunavut – with over 85% of the population indigenous, there is a wide gap in health status between these regions and Denmark and Canada; and
- the Russian Arctic – while the regions in the European North tend to fare better than those in Siberia, in almost any health indicator, the Arctic regions of Russia tend to occur at the lower end of the spectrum.

What are some of the health problems where disparities among regions are the greatest? Looking at the period 2005–2009, infant mortality rate ranges from 2 per 1,000 live births in Iceland to 28 in Koryakia. The mean national incidence rates for tuberculosis in the Nordic countries, Canada and the United States are less than 10/100,000. The rates for Greenland (130) and Nunavut (150) are more than 10 times higher. While the mean rate of the northern Russian regions is about 80, among them is Koryakia, with a rate as high as 450. Suicide among youth is particularly rampant in Greenland and Nunavut, a phenomenon observed also among Alaska Natives.

“Social determinants of health” is currently the dominant explanatory model for health disparities. To a large extent it is also applicable in the Arctic. Ecological studies confirm the association between socioeconomic indicators such as education and health outcomes, and life expectancy and infectious disease incidence. A social gradient exists not just for death and diseases, but also for behaviors such as smoking and alcohol abuse. Large population surveys across the Arctic, most notably the Survey of Living Conditions in the Arctic (SLiCA), provide some of this evidence. Moreover one needs to look beyond individual differences to explain variation at the population level. Historical, contextual and policy differences likely play a significant role in accounting for health disparities across circumpolar regions. Here is a ripe area not just for empirical research, but also for sound theoretical understanding and fresh insights.

## Health care challenges

Many of us are involved in circumpolar health in a variety of roles, as researchers, health care providers, administrators and policy makers. Health care may not solve all our problems, but it is amenable to innovations and improvements, and thus deserves our attention whatever our professional designation may be.

First, another reality check. As I have already mentioned, the North is highly urbanised. In the large urban centres, health care would not be very different from the southern parts of the country. It is not this “sector” where serious problems of access and quality arise, although issues of access and quality are national problems also. Thus a discussion of the challenges to health care in the North is really about that segment of the population living in remote, scattered, small villages and hamlets, exposed to the harsh environment and its adverse effects on infrastructure, facilities, equipment, and the quality of life and well-being of health care staff and patients. It is not just an issue of insufficient resources, since on a per capita level, many northern regions have health expenditures that are among the highest in the world. Whether they are “enough” or represent “value for money” is of course another issue, for which there are no easy answers. What is unclear is whether northern health care will soon reach, or has already reached, the point of unsustainability. Here is another research gap that remains to be filled.

Strategies are needed for health human resources development. Northern regions can build on many proud achievements – Alaska's health aides, Canada's primary care nurses, and Russia's feldschers and mobile medical teams come to mind – and should freely borrow best practices from one another. Multidisciplinary teams are a noble concept, that is, strong in rhetoric but instances where they are truly practised are few and far between. Much progress has also been made in bringing training of health professionals to the North.

I have come full circle to recognising the importance of technology innovations in remote health care. If developing countries can “leapfrog” technologies, surely Arctic countries, which are among the world's technologically most advanced countries, can develop and deploy technologies that can overcome existing barriers to equitable and effective health care? Just as mobile telephones have thrived in countries with crumbling conventional telecommunication infrastructure, why not use robots to replace the revolving door of short-term visiting health providers and free from the vagaries of travel due to uncooperative weather? Again, the North has taken the lead in testing telehealth technologies, but the time has surely come when we move beyond pilot studies to system change.

Although “knowledge translation” (KT) has acquired the status of a buzz word, it is safe to say that only a minority of devotees know what it really means and understand its intricacies. We would all agree that it is something that is needed. Researchers are rightly exhorted to engage in KT to justify the public money that has been showered upon them. Equally important, policy makers also need to recognise the importance of “evidence”, which is something that health researchers can provide.

## Looking ahead

A look at the congress program reassures me that circumpolar health is healthy and sound, and that at each congress there is a new crop of young recruits, with a few old-timers hanging around purportedly to provide “mentorship.” The circumpolar health “community” needs to be active in different fronts. Among Arctic scientists – the biologists, geologists, climatologists, etc. – health scientists are gaining increasing recognition, in part due to the prominence of health research in the activities of the International Polar Year. The momentum needs to be sustained. On another front, we need to keep on top of the political agenda of national and regional governments, and not just agencies involved in health care. At the international level, the Arctic Council is an important forum, and the creation of its Arctic Human Health Expert Group is a step in the right direction.

Perhaps it is a sign that circumpolar health has grown that we now have multiple organisations, consisting of more or less the same people, competing for the same small pool of resources. Circumpolar health is more than just the congress, which creates excitement once every 3 years, only to dissipate in the intervening interval. There needs to be on-going engagement of the “community.” There is tremendous opportunity for strong and sustained, rather than sporadic and intermittent, collaboration at the institutional and individual levels. Let us take advantage of this congress to network, engage in friendly debate and reach consensus.

## Further readings

This paper is unadorned – and thus uninterrupted – by footnotes and references. For further exploration of some of the ideas presented here, please consult:

1. Young TK, Bjerregaard P, eds. *Health transitions in Arctic populations*. Toronto: University of Toronto Press; 2008.
2. Young TK, Rawat R, Dallmann W, Chatwood S, Bjerregaard P, eds. *Circumpolar health atlas*. Toronto: University of Toronto Press; 2012.
3. Chatwood S, Bjerregaard P, Young TK. Global health – circumpolar perspectives. *Am J Public Health*. 2012;102:1246–9.

For statistical data, please visit the Circumpolar Health Observatory http://circhob.circumpolarhealth.org

